# “Clip-Stone” Filiation Within the Biliary Tract

**DOI:** 10.1155/1993/35965

**Published:** 1993

**Authors:** B. Mansvelt, J. Harb, B. Farkas, M. Mourou, C. Huguet

**Affiliations:** 1 Department of Surgery Princess Grace Hospital Principality of Monaco Monaco; 2 Department of Radiology Princess Grace Hospital Principality of Monaco Monaco

## Abstract

A case of cholangitis due to the migration of a metal clip used for surgical cholecystectomy 4 years earlier, is reported. The diagnostic approach and therapeutic options, either endoscopic or surgical are discussed. The use of resorbable clips during the performance of laparoscopic cholecystectomy should avoid this type of complication.


*HPB Surgery*, 1993, Vol. 6, pp. 185–188
Reprints available directly from the publisher
Photocopying permitted by license only

© 1993 Harwood Academic Publishers GmbH
Printed in the United States of America

### "CLIP-STONE" FILIATION WITHIN THE BILIARY TRACT

B. MANSVELT[1], J. HARB[1], B. FARKAS[2], M. MOUROU[2] and C. HUGUET[1]
*Departments of Surgery[1] and Radiology[2], Princess Grace Hospital, Monaco, Principality of Monaco*

*(Received 24 February 1992)*

A case of cholangitis due to the migration of a metal clip used for surgical cholecystectomy 4 years earlier, is reported. The diagnostic approach and therapeutic options, either endoscopic or surgical are discussed. The use of resorbable clips during the performance of laparoscopic cholecystectomy should avoid this type of complication.

KEY WORDS: Clip-stone, biliary tract

Although rare, the accidental migration of foreign bodies into the biliary tract is well known. Parasites (ascaris[1], fasciola hepatica[2], clonorchis sinensis[3] and non absorbable suture material used in a previous biliary tract procedure[4]) are the most common causes of bile duct obstruction. Metallic clips may also migrate from the cystic duct into the common bile duct and form the nidus for a stone, as we report here.

*Case*: A 88 year-old man was admitted for cholangitis four years after surgical cholecystectomy performed in another institution. Common bile duct obstruction was diagnosed with abdominal pain, jaundice, mild fever and cholestasis (bilirubin: Normal × 6, alkaline phosphatase: Normal × 2.5, Amylase: Normal × 20). Plain film of the abdomen revealed several clips in the right upper quadrant with one of them in front of L2 vertebra and which proved to be mobile on a control X-ray. Abdominal ultrasound demonstrated mild pancreatitis while the CT scan performed two days later, revealed a dilated common bile duct with metallic material in its lumen, attributable to a clip (Figure 1). Intravenous cholangiography confirmed the presence of a stone surrounding a metal clip at the lower end of the bile duct (Figure 2). In the absence of an experienced endoscopist, surgery was undertaken and the stone with the metal nidus could be easily extracted through the supra-duodenal bile duct. Four other clips surrounded with inflammatory granulomas were found close to the remnant cystic duct, suggesting they would have been eliminated in the same way as the first one. Complete excision of the cystic duct with the remaining clip and closure of the common duct with a continuous suture of absorbable material completed the procedure. The post operatory course was uneventful.

Address correspondence to: Professor Claude Huguet, M D, Department of Surgery, Princess Grace Hospital, Monaco.

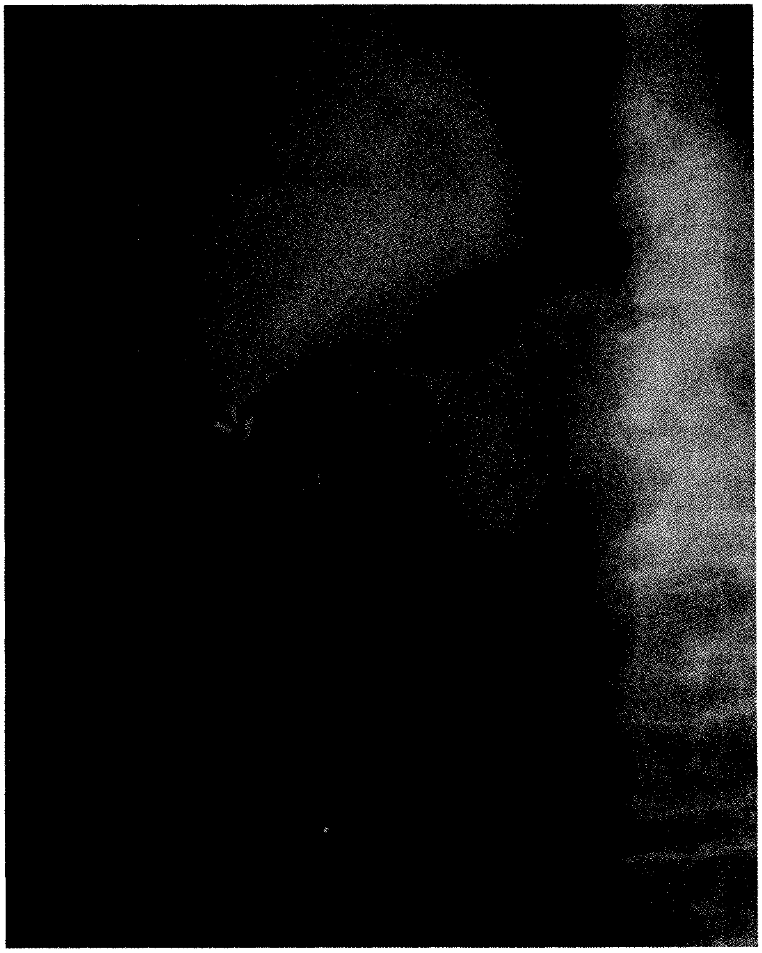

**Figure 1** CT Scan: Presence of metallic foreign body in the common bile duct.

### *Comments*

Homans[4] was the first, in 1897, to describe the formation of a common duct stone around a silk suture. In 1971 Ban *et al.*[5] reviewed 63 published cases of foreign bodies divided in three etiologic groups: (1) post operative, (2) penetrating trauma injury, and (3) alimentary material[5-8]. The most frequent cause is non resorbable suture material[9] followed exceptionally by a segment of a T tube[10-11] or of a gauze swab[12]. Metal clips have previously been reported by Walker[13], Brutvan[14], Von Wittenberg[15] and Margolis[16]. The clinical features are very suggestive with cholangitis and radiological evidence of intrabiliary metallic material. Following cholecystectomy, the delay of migration and stone formation varies from one to 13 years. When available, interventional endoscopy may be the treatment of choice[7-9-11-12], possibly associated with extracorporeal lithotripsy[17]. In case of multiple clips,

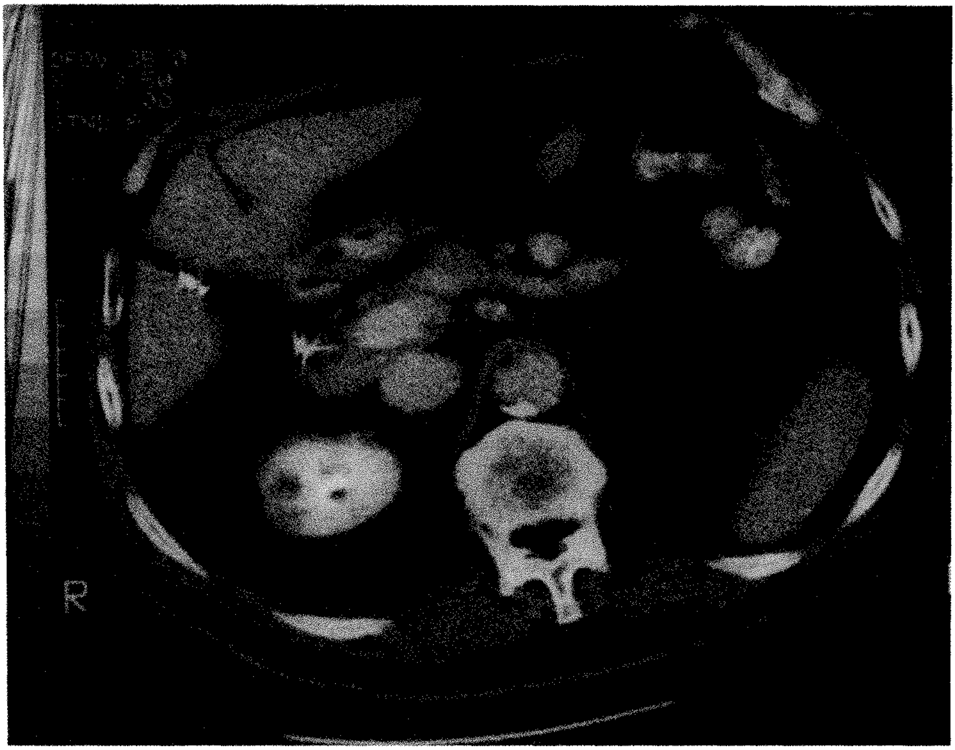

**Figure 2** Intravenous cholangiography. The stone surrounding a metal clip at the lower third of the bile duct.

surgery may be more suitable to avoid recurrent obstruction. Since laparoscopic cholecystectomy has become very popular today with routine application of metallic clips[18-20], other accidents will appear certainly in the future. The risk should be obviated by the use of resorbable clips which are already available for laparoscopic surgery.

### *References*

1. Estrada, J. and Garcia, E. (1942) Ascaris lumbricoides in the common bile duct. *J. Trop. Med.*, **45**, 33–36
2. Manson-Bahr, P. and Walton, J. (1940) The surgical removal of Fasciola hepatica from the common bile duct. *Br. J. Surg.*, **28**, 380–383
3. Sher, L., Iwatsuki, S. and Lebeau, G. (1989) Hilar cholangicarcinoma associated with clonorchisis. *Dig. Dis. Sci.*, **34**, 1121
4. Homans, J. (1897) Gall-stones formed around silk sutures, twenty months after recovery from cholecystectomy. *Ann. Surg.*, **26**, 114–116
5. Ban, J., Hirose, F. and Benfield, J. (1972) Foreign bodies of the biliary tract: report of two patients and a review of the literature. *Ann. Surg.*, **176**, 102–107
6. Leman, G., Lorré, A., Nelis, Ph., Verhofstadt, J., Badisco, K., Klerckx, P. and Van Mulders, P. (1990) Common bile duct stone caused by foreign body: a case report and literature review. *Acta Chir. Belg.*, **90**, 163–165

7. Orda, R., Leviav, A., Ratan, I., Stadler, J. and Wiznitzer (1986) Common bile duct stone caused by a foreign body. *J. Clin. Gastroenterol.*, **8**(4), 466–468
8. Morse, L. and Millin, J. (1971) Gallstone formation secondary to a foreign body. *The New England Journal of Medicine*, **284**, 590--591
9. Ormann, W. (1989) A thread as a nidus of a common bile duct calculus-finding during endoscopic lithotripsy. *Endoscopy*, **21**, 191–192
10. Banez, V., Leung, J. and Lau, W. (1990) Endoscopic management of an unusual intrabiliary foreign body. *Br. J. Surg.*, **77**, 882
11. Rüdiger, E., Weiss, E. and Schludermann, W. (1985) Letters to the editors: Endoscopic removal of the residual T-tube. *Endoscopy*, **17**, 85
12. Sakai, P., Ishioka, S., Zambrano, M., Marone, P. and Zitron, C. (1990) Endoscopic removal of an atypical common bile duct foreign body. *Endoscopy*, **22**, 93
13. Walker, W., Avant, G. and Reynolds, V. (1979) Cholangitis with a silver lining. *Arch. Surg.*, **114**, 214–215
14. Brutvan, F., Kampschroer, B. and Parker, H. (1982) Vessel clip as a nidus for formation of common bile duct stone. *Gastrointestinal Endoscopy*, **28**, 222–223
15. Von Wittenberg, H., Freise, J., Meyer, H. und Then, P. (1985) Gallensteinbildung nach transmuraler einwanderung eines operationsclips in die gallenwege. Kasuistik. *Z. Gastroenterologie*, **23**, 139–142
16. Margolis, J. (1986) Recurrent choledocholithiasis due to hemostatic clip. *Arch. Surg.*, **121**, 1213
17. Merrett, M. and Desmond, P. (1990) Removal of impacted endoscopic basket and stone from the common bile duct by extracorporeal shock waves. *Endoscopy*, **22**, 92
18. Dubois, F., Berthelot, G. and Levard, H. (1990) Cholécystectomie sous coelioscopie. *Annales de Chirurgie*, **44**, 203–206
19. Nathanson, L., Shimi, S. and Cuschieri, A. (1991) Laparoscopic cholecystectomy: the Dundee technique *Br. J. Surg.*, **78**, 155–159
20. Perissat, J., Coolet, D. and Belliard, R. (1990) Gallstones: laparoscopic treatment-cholecystectomy and lithotripsy. *Surg. Endosc.*, **4**, 15–17

*(Accepted by S. Bengmark 20 June 1992)*

### INVITED COMMENTARY

Foreign bodies in the common bile duct are relatively rare. The most common cause being residual objects from previous operations. Other causes are penetrating missile fragments or bullets and more rarely, ingested foreign bodies.

In our present era of the increased performance of laparoscopic cholecystectomies, we see a more common usage of silver clips for both cystic duct and cystic artery closure. The present report should alert us of the necessity to develop and use absorbable clips.

Prof. R. Orda
Assaf Harofeh Medical Center
Sackler Faculty of Medicine
Tel-Aviv University
Israel

